# Supplementary figures and images for: The dimeric Golgi protein Gorab binds to Sas6 as a monomer to mediate centriole duplication
Source: eLife. 2021 Mar 11;10:e57241. doi: 10.7554/eLife.57241 (PMC8009671; doi:10.7554/eLife.57241)

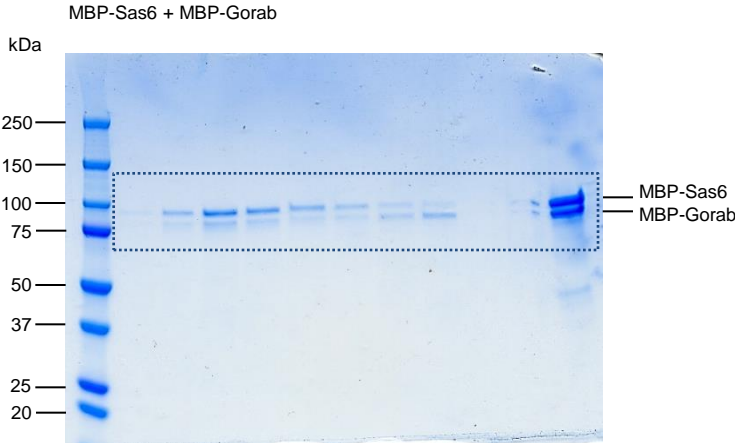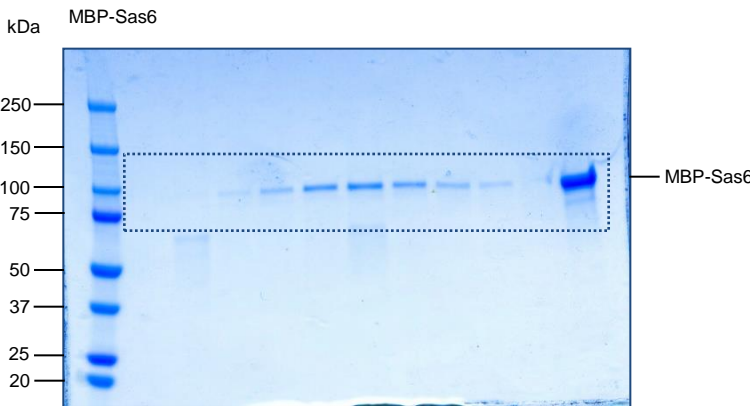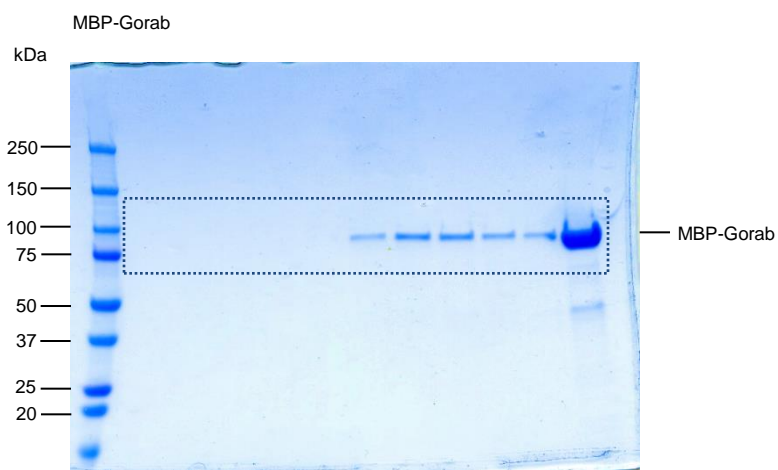

Supplement: Figure 2—source data 1. — Cropped SDS-PAGE fragments taken to the figure panel are shown. [file elife-57241-fig2-data1.pdf]

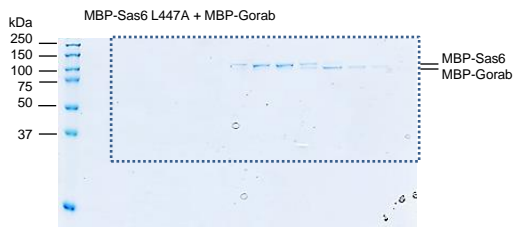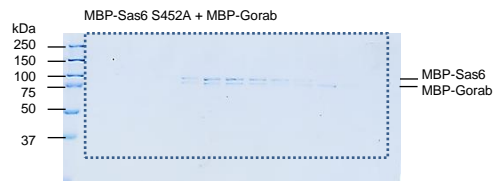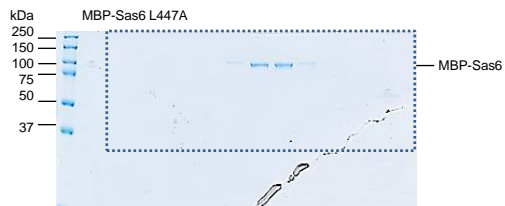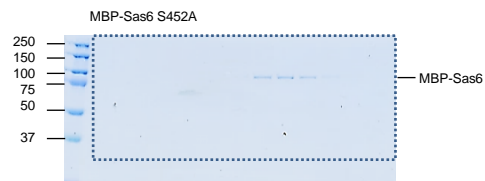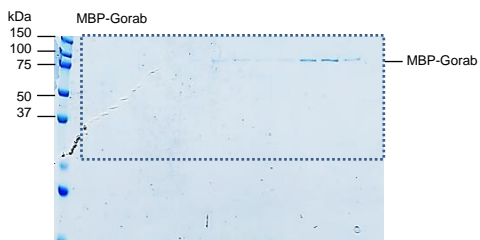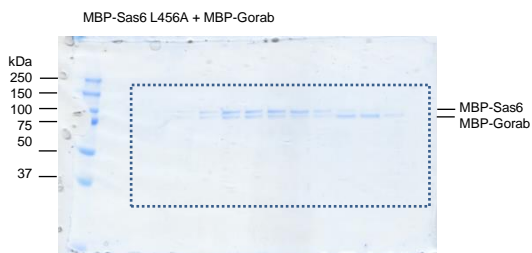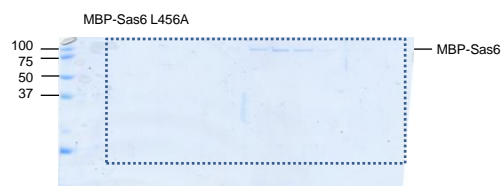

Supplement: Figure 3—figure supplement 2—source data 1. — Cropped SDS-PAGE fragments taken to the figure panel are shown. [file elife-57241-fig3-figsupp2-data1.pdf]
